# β-hydroxybutyrate Alleviates Learning and Memory Impairment Through the SIRT1 Pathway in D-Galactose-Injured Mice

**DOI:** 10.3389/fphar.2021.751028

**Published:** 2021-11-22

**Authors:** Xiaojing Yang, Ruonan Wang, Hailun Zhou, Li Wang, Rui Wang, Haomin Li, Baodong Tan, Qiong Wu, Xin Xu, Lianxu Cui, Zaiyu Li, Hua Li

**Affiliations:** ^1^ College of Pharmacy, Dalian Medical University, Dalian, China; ^2^ National-Local Joint Engineering Research Center for Drug-Research and Development (R & D) of Neurodegenerative Diseases, Dalian Medical University, Dalian, China; ^3^ Dalian Key Laboratory of Hematology, Department of Hematology, the Second Hospital of Dalian Medical University, Institute of Stem Cell Transplantation of Dalian Medical University, Dalian, China; ^4^ Department of Neurosurgery, Foshan Affiliated Hospital to Sun Yat -Sen University and the First People’s Hospital of Foshan, Foshan, China

**Keywords:** β-hydroxybutyrate, learning and memory impairment, oxidative stress, NLRP3, SIRT1, FoxO3a

## Abstract

Learning and memory impairment is a common clinical symptom of aging and nervous system injuries, and seriously affects quality of life. Memory impairment is associated with increased oxidative stress (OS) and inflammatory response. β-hydroxybutyrate (BHBA) is a water-soluble endogenous small-molecule ketone body that easily crosses the blood-brain barrier and has shown neuroprotection activities. In this study, we investigated the effects and mechanisms of BHBA on D-galactose (D-gal)-induced memory impairment in mice by *in vitro* and *in vivo* experiments. BHBA was administered intragastrically to D-gal-injured C57BL/6 mice for 42 days. Water maze performance, the morphology of the hippocampus with Nissl staining, the ACh content, OS, and inflammation status were examined. To further investigate the mechanism, hippocampal neuronal cells (HT22) were treated with BHBA with or without the SIRT1 inhibitor or small interfering RNAs against sirt1 (si-SIRT1) before incubation with D-gal. BHBA significantly improved water maze performance; increased the ACh content, SOD activity, and SIRT1 expression; and decreased AChE and LDH activity, ROS, MDA, IL-1β, TNF-α contents, and NLRP3 expression. Further studies with the SIRT inhibitor or siRNAs against sirt1 reversed the above effects of BHBA. Collectively, BHBA inhibited hippocampal OS and the inflammation process to alleviate learning and memory impairment through activating the SIRT1 pathway in D-gal-injured mice, suggesting that BHBA could be a potential option for drug development of learning and memory impairment induced by nervous system injuries.

## Introduction

Learning and memory function is one of the main functions of the brain. Learning and memory disorder is one of the main manifestations of aging. It is also one symptom of neurological disorders such as Parkinson’s disease (PD), Alzheimer’s disease (AD), vascular dementia (VD), and other neurologic damages by hypertensive cerebral hemorrhage, cerebral thrombosis, operation, and trauma (Zhao et al., 2017; Liu et al., 2018). How to effectively prevent and treat memory dysfunction caused by aging and neurological injuries has become one of the hot issues in the field of life and health in the world.

At present, only a few medications are used to improve memory function in clinic, such as memantine and donepezil with certain effects. Therefore, more novel and effective agents with fewer adverse effects need be developed. Previous studies have demonstrated that oxidative stress (OS) and neuroinflammation can lead to neuronal loss or permanent functional changes (Allison and Ditor, 2014; Nickels et al., 2016; Zhao et al., 2017) and produce memory impairment (Yan et al., 2018). Sirtuin 1 (SIRT1) is one member of the sirtuins family, which is a family of proteins with homologies to the silence information regulator 2 (SIR2) gene of yeast (Brachmann et al., 1995). SIRT1, an NAD+-dependent deacetylase, is widely distributed in the brain and plays important roles in suppressing inflammatory responses, anti-OS, and antiaging, and regulating autophagy and apoptosis. Forkhead box O transcription factors (FOXO) are a subgroup of Forkhead box (FOX) transcription factors that are involved in many physiological and pathological processes of the body (Carter and Brunet, 2007). FOXO3A is an important member of the FOXO family, which inhibits tumor growth and OS, induces apoptosis, and regulates the cell cycle (Bullock, 2016; Gunnarsdóttir et al., 2020). Studies showed that FOXO3A was deacetylated by SIRT1 to induce OS in bovine aortic endothelial cells (Olmos et al., 2013). *Astragalus* Bai Ping Lung Capsules produced anti-OS and anti-inflammatory effects by regulating the SIRT1/FOXO3A pathway (Wu et al., 2019), which in turn effectively alleviated the symptoms of rats suffering from chronic obstructive pulmonary disease. The above research studies suggest that SIRT1 and FOXO3A act as upstream active factors to regulate OS and inflammation processes, and drugs activating SIRT1 have the potential for the prevention and therapy of memory dysfunction.

β-hydroxybutyrate (BHBA), with a molecular weight of 104.1Da, is a kind of water-soluble ketone body. BHBA easily crosses the blood-brain barrier (BBB) and provides energy for the brain instead of glucose in the hypoglycemic state. Previous studies have found that BHBA also served as an important signaling molecule to maintain normal neurological functions. It suppressed LPS-induced inflammation *via* inhibiting NF-κB activation in mice microglia cells (Fu et al., 2014) and attenuated neuronal damage in epileptic mice (Guo et al., 2019). In addition, BHBA inhibited neurodegenerative processes in Cockayne syndrome (CS). It ameliorated the DNA repair protein CS group B by upregulating SIRT1 and inhibiting the sustained activation of poly-ADP-ribose polymerase 1 (PARP1) (Scheibye-Knudsen et al., 2014). BHBA also significantly reduced renal ischemia-reperfusion injury by inducing FOXO3, decreasing the expression of caspase-1, interleukin-1β (IL-1β), and interleukin-18 (IL-18) (Tajima et al., 2019). These results prompted opinion that BHBA may play a role in improving learning and memory function through activating the SIRT1 pathway.

Therefore, the present study aimed to investigate the effects of BHBA on learning and memory impairment with a D-gal-induced injury model and to determine whether SIRT1 mediates these effects through regulating OS and inflammation processes.

## Materials and Methods

### Chemicals

Both sodium β-hydroxybutyrate (sodium salt of BHBA) and donepezil were purchased from Shanghai Xian Ding Biotechnology Co. D-galactose was purchased from Beijing Solabao Technology Co. The analytical purity of all the chemicals used in this study was above 98%.

### Animal Experiments

SPF-grade healthy male C57BL/6 mice (6–8 weeks), weighing 16–20 g, were obtained from the Dalian Medical University Animal Center (Dalian, China). All procedures were performed in accordance with institutional guidelines for animal use and care and approved by the Institutional Ethics Committee (IACUC number: L2013011). The mice were acclimatized for 1 week prior to the experiment according to a 12 h light–dark cycle in an environment with a relative humidity of 40–70% and a relative temperature of 25°C. Clean water, experimental animal feed, and bedding were given, and they were allowed to feed and drink freely. Seventy C57BL/6 mice were randomly divided into the following seven groups (n = 10): control (PBS, i.g), BHBA (500 mg/kg, i.g), D-gal (180 mg/kg, i.p), D-gal + BHBA (125 mg/kg, i.g), D-gal + BHBA (250 mg/kg, i.g), D-gal + BHBA (500 mg/kg, i.g), and D-gal + donepezil (3 mg/kg, i.g). The doses were administered to the mice once a day for 42 days. All drugs were dissolved in PBS solution. Mice were subjected to the water maze test at the end of drug administration. After the water maze test, the mice in each group were weighed and anesthetized. Hippocampal tissue samples were taken and stored according to the different assays: histologically stained specimens were fixed using 4% paraformaldehyde solution and stored at room temperature; the rest of the hippocampal tissues were stored frozen at −80°C.

### HT22 Cell Culture

The mouse hippocampal neuronal cells (HT22) (Sebacon Biotechnology, China) were cultured in Dulbecco’s modified Eagle’s medium (DMEM) (Hyclone, United States) in a constant temperature incubator (ThermoFisher Scientific, United States) at 37°C, 5% CO_2_, and saturated humidity. Treatment was performed according to the following groups: control, BHBA (20 mM), D-gal (200 mM), D-gal + BHBA (5 mM), D-gal + BHBA (10 mM), D-gal + BHBA (20 mM), D-gal + donepezil (2 μM). BHBA, donepezil, D-galactose, and equal amounts of the blank solvent were applied separately according to the groups for 24 h, and the cells or culture medium were collected. All drugs were dissolved in serum-free DMEM.

In the experiments with SIRT1 inhibitor EX527, HT22 cells were divided into five groups: control, BHBA (20 mM), D-gal (200 mM), D-gal + BHBA (20 mM), and D-gal + BHBA (20 mM) + EX527(1 μM). EX527 was added 4 h before cell administration, followed by subsequent manipulations.

In the experiments with si-SIRT1 inhibition, HT22 cells were divided into five groups: control, BHBA (20 mM), D-gal (200 mM), D-gal + BHBA (20 mM), and D-gal + BHBA (20 mM) + si-SIRT1. si-SIRT1 (5′-3': GCA​CCG​AUC​CUC​GAA​CAA​UTT; 5′-3': AUU​GUU​CGA​GGA​UCG​GUG​CTT) was transfected with lipo3000 for 24 h, followed by subsequent operations.

### The Water Maze Test

The water maze test is used to test the learning and memory ability of mice. The device is divided into four quadrants of equal sizes. Briefly, during the navigation test, a circular escape platform was placed 1 cm underwater in the middle of a fixed quadrant, and mice were randomly placed in one of the quadrants and allowed to swim until they found the platform or for 60 s. If the mice could not find the platform, they were guided to the platform for 5 s. The mice were given a rest for 5 min between training sessions. All mice were trained for 4 days, and the tracking software (NoldusEtho Vision system, version 5, Everett, WA, United States) was used on the fifth day to automatically record the swimming speed and escape latency. On the sixth day, a spatial probe test was performed simply by removing the platform and allowing the mice to swim freely for 60 s. The crossing frequency of traveling through the platform area was recorded.

### Nissl Staining

Hippocampal tissues were fixed with 4% paraformaldehyde solution. After dehydration, embedding, and sectioning, samples were stained according to the Nissl staining instructions (Solebro, China) and observed under an orthomosaic microscope (Leica, Germany).

### ACh, AChE, LDH, SOD, and MDA Kit Assay

Contents of acetylcholine (ACh), malondialdehyde (MDA), activities of lactate dehydrogenase (LDH), superoxide dismutase (SOD), and acetylcholinesterase (AChE) were measured using assay kits (ACh, MDA, LDH, and AChE kits: Nanjing Jiancheng Company, China; SOD kit: Beyotime Institute of Biotechnology, China). The absorbance was detected using an EnSpire microplate reader (PerkinElmer, United States).

### Intracellular ROS Assay

The 2,7-dichlorodihydrofluorescence indiacetate (DCFH-DA) ROS probe (Beyotime Institute of Biotechnology, China) was used to detect intracellular ROS. The cells were observed and photographed using a fluorescence microscope (Nikon, Japan), and the fluorescence intensity of intracellular ROS was analyzed using Image J analysis software (NIH, United States).

### IL-1β and TNF-α ELISA Kit Assay

IL-1β and tumor necrosis factor-α (TNF-α) were detected with IL-1β (Proteintech Group, INC., China) and TNF-α (KeyGEN BioTECH, China) ELISA kits according to the manufacturer’s recommendations, and the absorbance was measured with an EnSpire ELISA (PerkinElmer, United States).

### Western Blot Analysis

The protein concentration in the hippocampus or cells was detected using a BCA kit (Biyuntian Institute of Biotechnology, China) and adjusted to the same level. The samples were separated by sodium dodecyl sulfate polyacrylamide gel electrophoresis (SDS-PAGE) and then transferred to a polyvinylidene PVDF membrane (Millipore, Bedford, MA, United States). After being blocked with 5% skim milk for 2 h, the membrane was incubated with primary antibodies overnight at 4°C and secondary antibodies for 2 h at room temperature. The specific primary antibodies were against SIRT1 (1:1,000, Proteintech Group, INC., China), FOXO3A (1:1,000, Proteintech Group, INC., China), and NLRP3 (1:1,000, Zen-Bioscience, China). The secondary antibodies were the horseradish enzyme-labeled goat antimouse secondary antibody (1:1,000, ZhongShan Jinqiao Biotechnology Co., Ltd., China) and the horseradish enzyme-labeled goat antirabbit secondary antibody (1:1,000, ZhongShan Jinqiao Biotechnology Co., Ltd., China). The protein bands on the membrane were recorded using a gel imaging system (UVP, California, United States).

### Statistical Analysis

Data are presented in the form of mean ± SEM. Data were analyzed with one-way analysis of variance (ANOVA) or a *t*-test using GraphPad Prism 8 statistical software (GraphPad, La Jolla, CA, United States). *p* < 0.05 indicates that the differences were statistically significant.

## Results

### Effects of BHBA on the Behavior of D-Gal-Induced Mice in the Water Maze Test

**FIGURE 1:**
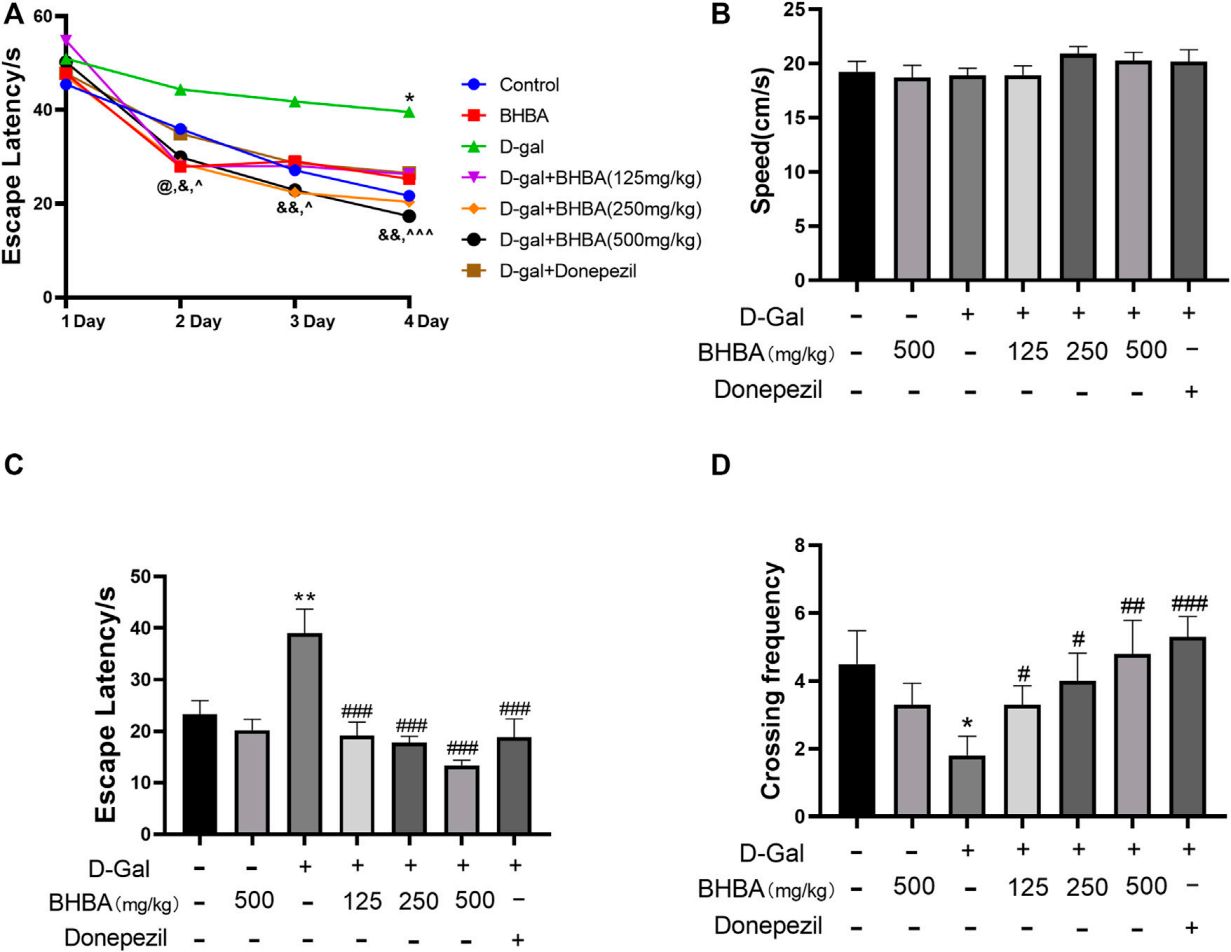
Effects of BHBA on behavior in the water maze test in D-gal-induced mice. **(A)** Escape latency during training. **(B)** Swimming speed on the fifth day. **(C)** Escape latency on the fifth day. **(D)** Crossing frequency on the fifth day. D-gal: 180 mg/kg; BHBA: 125, 250, and 500 mg/kg, respectively; donepezil: 3 mg/kg, n = 8–10. Compared with the control group, *p < 0.05, **p < 0.01; compared with the D-gal group, #*p* < 0.05, ##*p* < 0.01, ###*p* < 0.001. D-gal + BHBA (125 mg/kg) compared with the D-gal group, @*p* < 0.05, D-gal + BHBA (250 mg/kg) compared with the D-gal group, &*p* < 0.05, &&*p* < 0.01, D-gal+BHBA (500 mg/kg) compared with the D-gal group, *^p* < 0.05, ^ ^ ^*p* < 0.001.

The water maze test is a classic behavioral experiment used to test learning and memory ability. As shown in Figure 1A, during the training days, the escape latency decreased gradually in all groups of mice. However, the D-gal group showed a significant higher escape latency on the fourth day compared with the control group. The BHBA treatment group showed a significantly reduced escape latency from the second day to the fourth day compared with the D-gal group. As shown in Figure 1B, on the fifth day, there was no significant difference in swimming speed among groups. As shown in Figures 1C,D, on the fifth day, the D-gal group showed a significant increase in escape latency and a significant decrease in crossing frequency compared with the control group, while the BHBA treatment group showed a significantly reduced escape latency and increased crossing frequency compared with the D-gal group.

### Effects of BHBA on D-Gal-Induced Hippocampal Damage, ACh, and AChE in Mice

**FIGURE 2:**
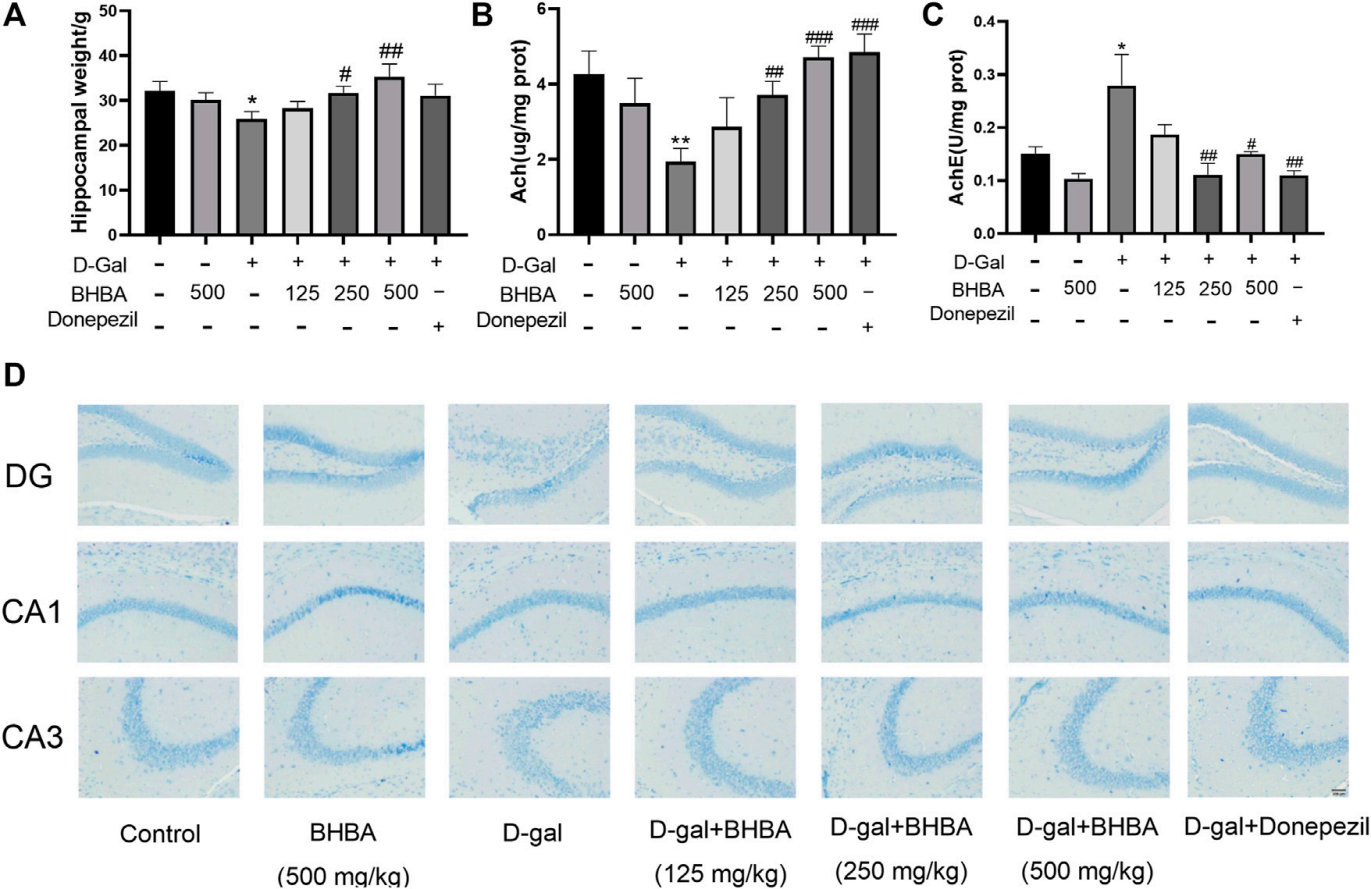
Effects of BHBA on hippocampal damage, ACh, and AChE in D-gal-induced mice. **(A)** Hippocampal weight. **(B)** Content of ACh. **(C)** Activity of AChE. **(D)** Hippocampal morphology with Nissl staining (magnification: 200X). D-gal: 180 mg/kg; BHBA: 125, 250, and 500 mg/kg, respectively; donepezil: 3 mg/kg, n = 8. Compared with the control group, *p < 0.05, **p < 0.01; compared with the D-gal group, #*p* < 0.05, ##*p* < 0.01, ###*p* < 0.001.

As shown in Figures 2A–C, compared with the control group, the hippocampal weight and the hippocampal ACh content were significantly decreased, and AChE activity was significantly enhanced in the D-gal group. BHBA treatment in D-gal-induced mice significantly reversed the above changes. As shown in Figure 2D, compared with the control group, the hippocampal Nissl bodies in the D-Gal group were disordered and sparse with blurred edges, while Nissl bodies in the D-Gal + BHBA groups were neatly arranged and dense with clear edges.

### Effects of BHBA on Hippocampal OS and Inflammation in D-Gal-Induced Mice

**FIGURE 3:**
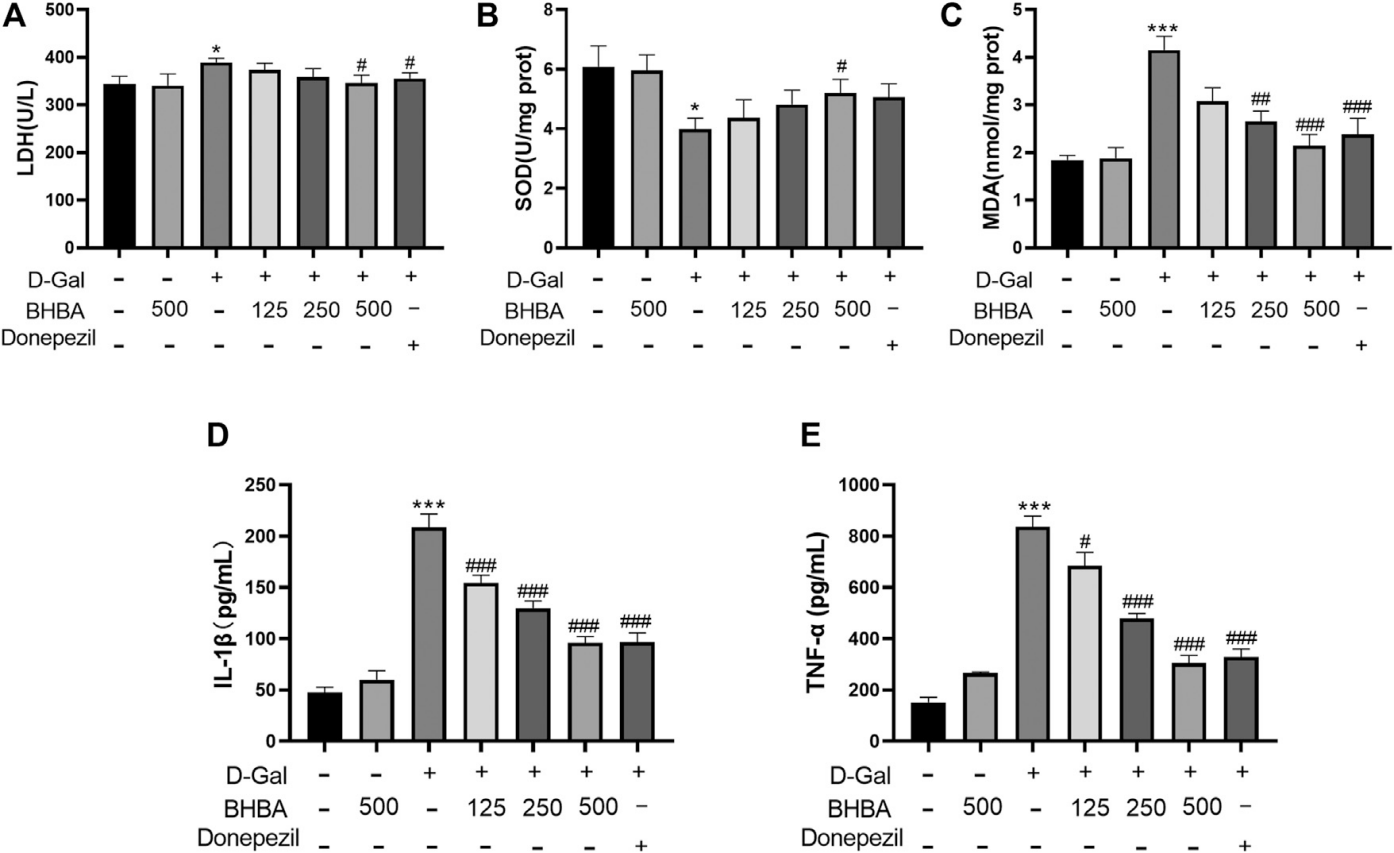
Effects of BHBA on hippocampal oxidative stress and inflammation in D-gal- induced mice. **(A)** Activity of LDH. **(B)** Activity of SOD. **(C)** Content of MDA. **(D)** Content of IL-1β. **(E)** Content of TNF-α. D-gal: 180 mg/kg; BHBA: 125, 250, and 500 mg/kg, respectively; donepezil: 3 mg/kg, n = 7–8. Compared with the control group, *p < 0.05, ***p < 0.001; Compared with the D-gal group, #*p* < 0.05, ##*p* < 0.01, ###*p* < 0.001.

As shown in Figures 3A–E, compared with the control group, the hippocampal LDH activity, MDA, IL1-β, and TNF-α contents were significantly enhanced, and the SOD activity was significantly weakened in the D-gal group. BHBA treatment, especially at a high dosage (500 mg/kg), significantly reversed the above changes in D-gal-injured mice.

### Effects of BHBA on Hippocampal SIRT1, FOXO3A, and NLRP3 in D-Gal-Induced Mice

**FIGURE 4:**
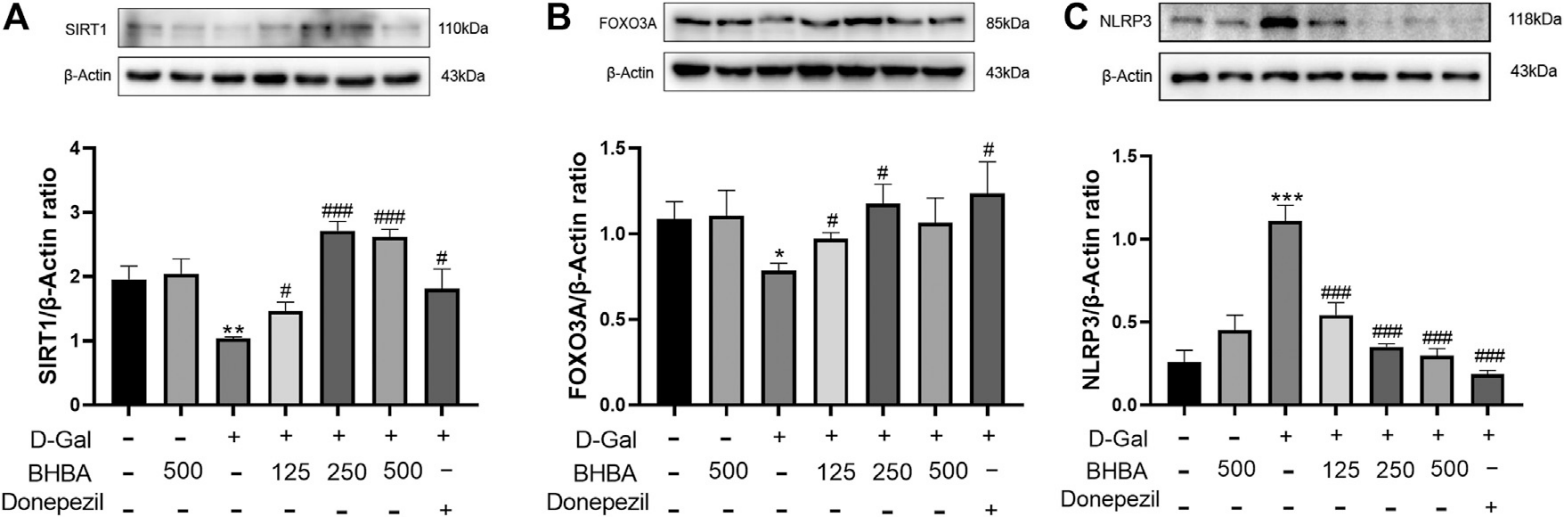
Effects of BHBA on hippocampal SIRT1, FOXO3A, and NLRP3 expression in D-gal-induced mice. **(A)** Protein expression of SIRT1. **(B)** Protein expression of FOXO3A. **(C)** Protein expression of NLRP3. D-gal: 180 mg/kg; BHBA: 125, 250, and 500 mg/kg, respectively; donepezil: 3 mg/kg, n = 3. Compared with the control group, *p < 0.05, **p < 0.01, ***p < 0.001; compared with the D-gal group, #*p* < 0.05, ###*p* < 0.001.

As shown in Figures 4A–C, compared with the control group, the hippocampal SIRT1 and FOXO3A expression was significantly reduced, and NLRP3 expression was significantly increased in the D-gal group, while BHBA treatment in D-gal-injured mice significantly reversed the above changes.

### Effects of BHBA on Oxidative Stress and Inflammation in D-Gal-Induced HT22 Cells

**FIGURE 5:**
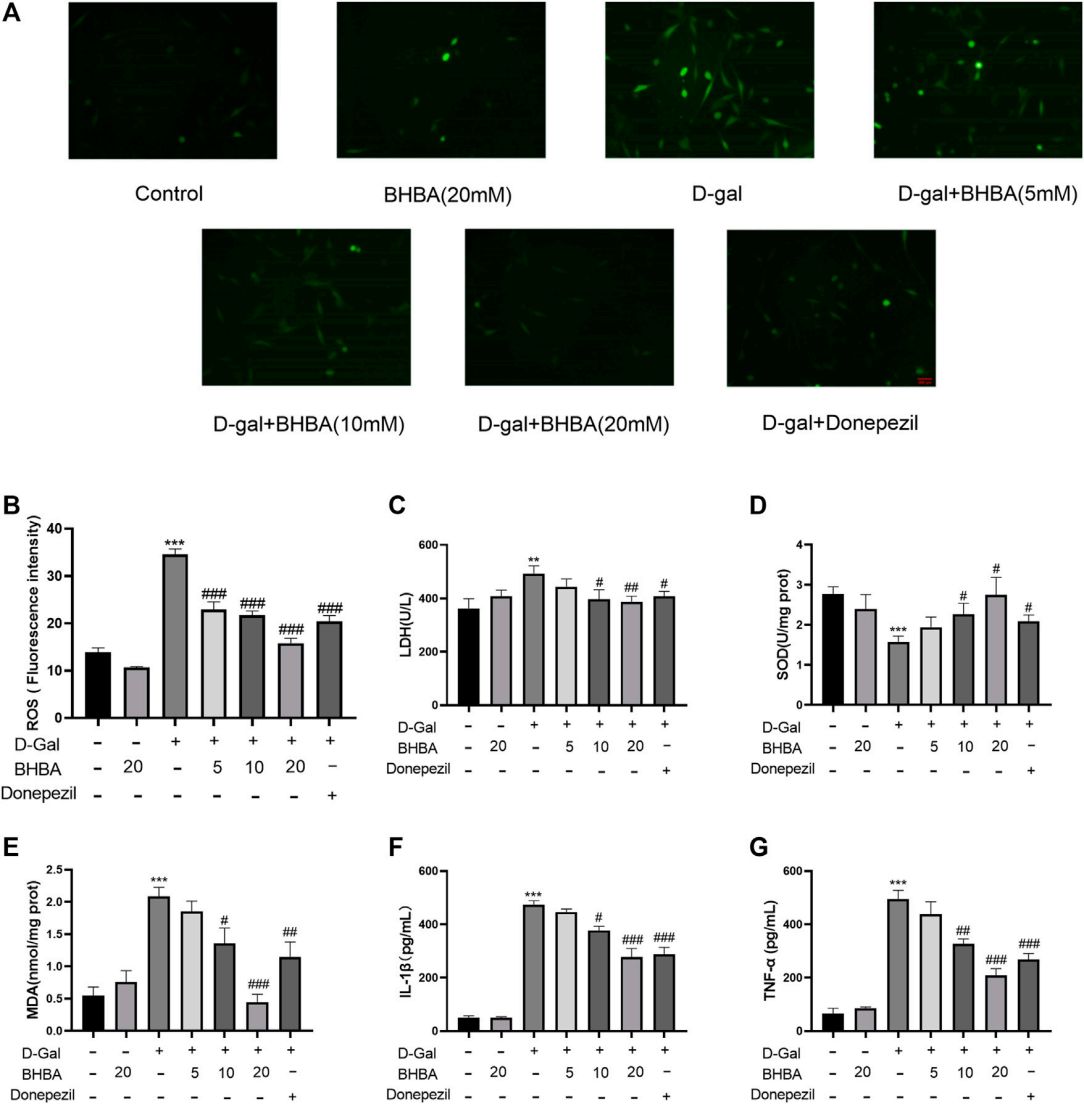
Effects of BHBA on oxidative stress and inflammation in D-gal-induced HT22 cells. **(A)** Fluorescence of ROS (magnification: 200X). **(B)** Fluorescence intensity of ROS. **(C)** Activity of LDH. **(D)** Activity of SOD. **(E)** Content of MDA. **(F)** Content of IL-1β. **(G)** Content of TNF-α. D-gal: 200 mM; BHBA: 5, 10, and 20 mM, respectively; donepezil: 2 μM, n = 3–6. Compared with the control group, **p < 0.01, ***p < 0.001; compared with the D-gal group, #*p* < 0.05, ##*p* < 0.01, ###*p* < 0.001.

As shown in Figures 5A–G, compared with the control group, the LDH activity, intracellular ROS fluorescence intensity, MDA, IL1-β, and TNF-α contents were significantly increased, and SOD activity was significantly reduced in the D-gal group. Compared with the D-gal group, BHBA treatment (10 and 20 mM) significantly reversed the above changes induced by G-gal.

### Effects of BHBA on the Expression of SIRT1, FOXO3A, and NLRP3 in D-Gal-Induced HT22 Cells

**FIGURE 6:**
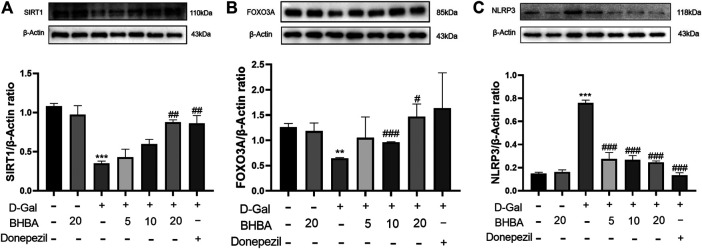
Effects of BHBA on the expression of SIRT1, FOXO3A, and NLRP3 in D-gal-induced HT22 cells. **(A)** Protein expression of SIRT1. **(B)** Protein expression of FOXO3A. **(C)** Protein expression of NLRP3. D-gal: 200 mM; BHBA: 5, 10, and 20 mM, respectively; donepezil: 2 μM, n = 3. Compared with the control group, *p < 0.05,**p < 0.01, ***p < 0.001; compared with the D-gal group, #*p* < 0.05, ##*p* < 0.01, ###*p* < 0.001.

As shown in Figures 6A–C, the expression of SIRT1 and FOXO3A was significantly decreased and the expression of NLRP3 protein was significantly increased in the D-gal group compared with the control group. Compared with the D-gal group, the expression of SIRT1 and FOXO3A protein was significantly increased in the D-gal + BHBA (20 mM) group and the NLRP3 protein expression was more significantly decreased after BHBA (5–20 mM) treatment.

### Influence of SIRT1 Inhibitor EX527 on the Effects of BHBA on D-Gal-Induced HT22 Cells

**FIGURE 7:**
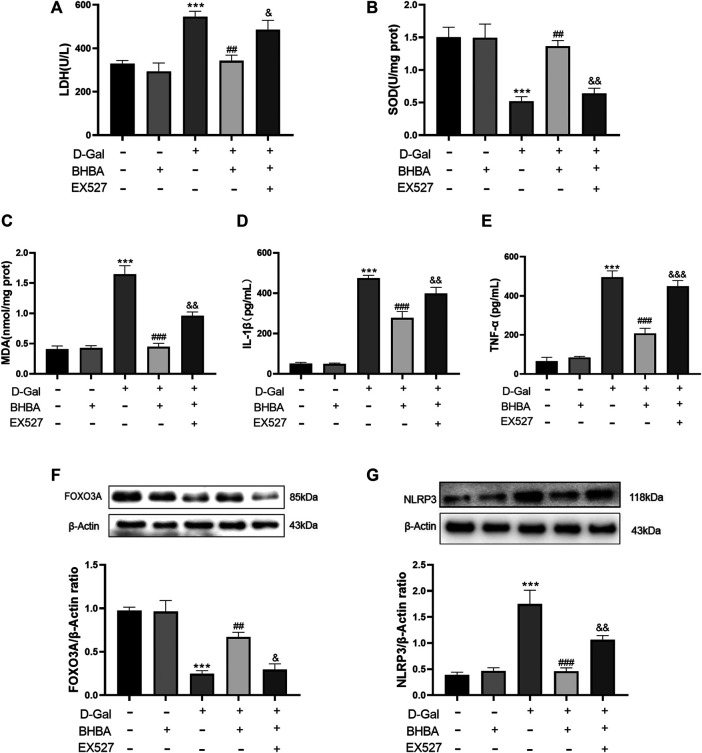
Influence of SIRT1 inhibitor EX527 on the effects of BHBA on D-gal-induced HT22 cells. **(A)** Activity of LDH. **(B)** Activity of SOD. **(C)** Content of MDA. **(D)** Content of IL-1β. **(E)** Content of TNF-α. **(F)** Protein expression of FOXO3A. **(G)** Protein expression of NLRP3. D-gal: 200 mM; BHBA: 20 mM; EX527: 1 μM, n = 3–5. Compared with the control group, ***p < 0.001; compared with the D-gal group, ##*p* < 0.01, ###*p* < 0.001; compared with the D-gal + BHBA group, &*p* < 0.05, &&*p* < 0.01, &&&*p* < 0.001.

As shown in Figures 7A–G, compared with the control group, LDH activity, MDA, IL1-β, and TNF-α levels were significantly increased, and SOD activity was significantly weakened in the D-gal group. FOXO3A protein expression was significantly decreased, and NLRP3 protein expression was significantly increased. Compared with the D-gal group, BHBA treatment at a high dosage (20 mM) significantly reversed the above effects induced by D-gal, while pretreatment with SIRT1 inhibitor EX527 obviously inhibited the effects of BHBA on D-gal-induced injuries.

### Influence of si-SIRT1 on the Effects of BHBA on D-Gal-Induced HT22 Cells

**FIGURE 8:**
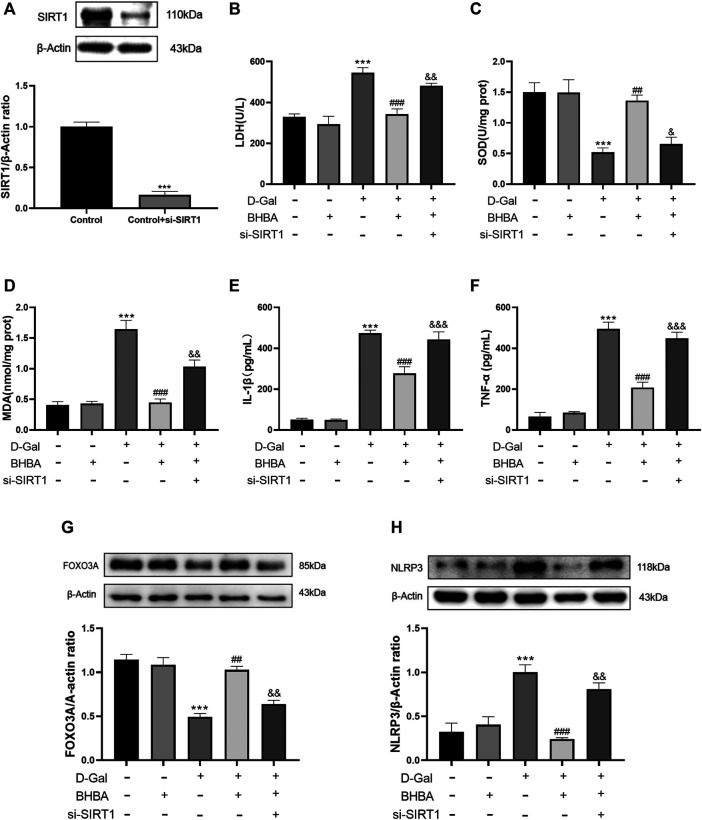
Influence of si-SIRT1 to the effects of BHBA on D-gal-induced HT22 cells. **(A)** Protein expression of SIRT1. **(B)** Activity of LDH. **(C)** Activity of SOD. **(D)** Content of MDA. **(E)** Content of IL-1β. **(F)** Content of TNF-α. **(G)** Protein expression of FOXO3A. **(H)** Protein expression of NLRP3. D-gal: 200 mM; BHBA: 20 mM, n = 3–5. Compared with the control group, ***p < 0.001; compared with the D-gal group, ##*p* < 0.01, ###*p* < 0.001; compared with the D-gal +BHBA group, &&*p* < 0.01, &&&*p* < 0.001.

As shown in Figure 8A, si-SIRT1 effectively inhibited the expression of SIRT1 protein. As shown in Figures 8B–H, the D-gal group showed obvious OS and an inflammation status compared with the control group. BHBA treatment at a high dosage (20 mM) significantly reversed the above effects induced by D-gal, while pretreatment with si-SIRT1 showed similar effects with the SIRT1 inhibitor, EX527, i.e., si-SIRT1 obviously weakened the effects of BHBA on D-gal-induced injuries.

## Discussion

The central nervous system is susceptible to oxidative damage and learning and memory impairments due to its high oxygen and unsaturated lipid contents and the relative lack of antioxidant defense mechanisms (Akbaraly et al., 2013). Studies indeed showed that the aging and neurological disorder-related impairments of learning and memory are gradually increasing and impose a heavy toll on patient’s quality of life and society. Therefore, it is urgent to develop more effective and safe drugs. Accumulated data strongly suggest that OS and neuroinflammation are among the main causes of neurodegenerative progress. Oxidative damage induces neuroinflammation, which is evidenced by the increased production of proinflammatory factors such as IL-1β and TNF-α. Furthermore, OS and inflammation promote each other and work together to accelerate the progress of learning and memory impairments and neurological injuries. Therefore, in the present study, the classic model of learning and memory impairments induced by D-gal was used since D-gal induced significant learning and memory impairments in mice by promoting OS and hippocampal injury (Zhao et al., 2017).

BHBA is a more potential option for neurological drug development since it possesses some properties, such as a small molecular weight (104.1Da), high penetrability of BBB, and cytoprotective activity. BHBA reduced β-amyloid protein (Aβ)-induced neuronal apoptosis in a rat model and activated the nuclear transcription factor Nrf2 to exert anti-OS effects in Aβ-injured PC12 cells (Xie et al., 2015). BHBA also induced the expression of the antioxidant enzyme, heme oxidase 1, and reduced inflammatory cytokine production, suggesting its anti-inflammatory and antioxidative effects (Miyauchi et al., 2019). However, there is still no published report on the study of BHBA on D-gal-induced memory deficits.

In order to investigate the effects of BHBA on D-gal-induced memory disorders in mice, the classic water maze test was used. BHBA significantly increased the number of shuttles and decreased the escape latency without influence on the swimming speed, indicating that BHBA improved learning and memory behavior, which was not caused by the improvement of motor function. The hippocampus, an important part of the limbic system, is the key site responsible for learning and memory function. The hippocampal cholinergic nervous system plays an important role in the learning and memory process. ACh is the main neurotransmitter for cholinergic transmission and the regulation of learning and memory function (Hampel et al., 2018). Massive cholinergic denervation in PD caused severe cognitive decline (Ray et al., 2018). Therefore, ACh content and AChE activity are common indicators for learning and memory function. In the present study, BHBA significantly increased ACh content and decreased AChE activity in the hippocampus of mice. BHBA also significantly increased the hippocampal weight and improved the morphology of hippocampal neurons, suggesting that BHBA improves the learning and memory function in mice by inhibiting hippocampal neuronal damage and hippocampal atrophy and improving hippocampal cholinergic dysfunction. Further studies showed that BHBA significantly reduced LDH activity, TNF-α, IL-1β, and MDA content, and enhanced SOD activity in the hippocampus. Further studies on HT22 cells, the mouse hippocampal neuronal cells, were consistent with the above results, suggesting that these effects of BHBA may be related to its inhibitory effects on hippocampal OS and neuroinflammation.

OS and inflammation are closely related pathophysiological processes. Both OS and inflammation alter the expression of IL1, FOXO, and TNF-α. SIRT1 has been shown to inhibit OS and inflammation in the brain. SIRT1 regulates the FOXO transcription factor by deacetylation to prevent neurodegeneration due to OS. Deletion of SIRT1 in the brain significantly increases the ROS and inflammatory responses (Singh and Ubaid, 2020). Nucleotide-binding oligomerization domain (NOD)-like receptor containing pyrin domain 3 (NLRP3) is the most fully characterized inflammasome which is involved in the amount of human inflammatory and autoimmune diseases. NLRP3 are upregulated and expressed by both glial and neuronal cells in sclerotic hippocampi from patients with temporal lobe epilepsy. NLRP3 inflammasome signaling triggers the production of the proinflammatory mediators IL-1β and then releases proinflammatory cytokines TNF-α (Cristina de Britotoscano et al., 2021). Activation of the NLRP3 inflammasome induces systemic chronic inflammation in aging. Ablation of the NLRP3 inflammasome protected mice from age-related increases in chronic inflammation and the innate immune activation (Youm et al., 2013; He et al., 2020). In the present study, BHBA treatment significantly reversed the downregulation of SIRT1 and FOXO3A and the upregulation of NLRP3 induced by D-gal *in vivo* and *in vitro*, which may explain the effects of BHBA on hippocampal OS and neuroinflammation. Since SIRT1 was generally accepted as the upstream active factor to regulate OS and inflammation, the inhibitor of SIRT1 or siRNAs against the sirt1 gene was used, respectively. The inhibition of SIRT1 reversed the above effects of BHBA on FOXO3A, NLRP3, and other OS and inflammatory indicators in D-gal-injured HT22 cells.

In conclusion, BHBA can improve cholinergic dysfunction and hippocampus injury to alleviate the learning and memory impairments induced by D-gal in mice. The mechanism is related to activating SIRT1 by BHBA, possibly leading to the upregulation of FOXO3A and downregulation of NLRP3 and then the inhibition of OS and inflammation in the hippocampus. The present study revealed that SIRT1 is a promising target for therapeutic intervention in learning and memory deficits and BHBA may be a potential candidate for development as a novel drug.

## Data Availability

The raw data supporting the conclusions of this article will be made available by the authors, without undue reservation.
